# The effect on behavior and bone mineral density of individualized bone mineral density feedback and educational interventions in premenopausal women: a randomized controlled trial [NCT00273260]

**DOI:** 10.1186/1471-2458-6-12

**Published:** 2006-01-23

**Authors:** Tania Winzenberg, Brian Oldenburg, Sue Frendin, Laura De Wit, Malcolm Riley, Graeme Jones

**Affiliations:** 1 Menzies Research Institute, University of Tasmania, Hobart, Australia; 2 School of Public Health, Queensland University of Technology, Brisbane, Australia; 3 Department of Health and Human Services, Government of Tasmania, Hobart, Australia; 4 Nutrition and Dietetic Unit, Monash University, Melbourne, Australia

## Abstract

**Background:**

Limited information is available on ways to influence osteoporosis risk in premenopausal women. This study tested four hypotheses regarding the effects of individualized bone density (BMD) feedback and different educational interventions on osteoporosis preventive behavior and BMD in pre-menopausal women, namely: that women are more likely to change calcium intake and physical activity if their BMD is low; that group education will be more efficacious at changing behavior than an information leaflet; that BMD feedback and group education have independent effects on behavior and BMD; and, that women who improve their physical activity or calcium intake will have a change in bone mass over 2 years that is better than those who do not alter their behavior.

**Methods:**

We performed a 2-year randomized controlled trial of BMD feedback according to T-score and either an osteoporosis information leaflet or small group education in a population-based random sample of 470 healthy women aged 25–44 years (response rate 64%). Main outcome measures were dietary calcium intake, calcium supplement use, smoking behavior, physical activity, endurance fitness, lower limb strength and BMD. We used paired t-tests, one-way ANOVA and linear regression techniques for data analysis.

**Results:**

Women who had feedback of low BMD had a greater increase in femoral neck BMD than those with normal BMD (1.6% p.a. vs. 0.7% p.a., p = 0.0001), but there was no difference in lumbar spine BMD change between these groups (0.1% p.a. vs. 0.08% p.a., p = 0.9). Both educational interventions had similar increases in femoral neck BMD (Leaflet = +1.0% p.a., Osteoporosis self-management course = + 1.3% p.a., p = 0.4). Femoral neck BMD change was only significantly associated with starting calcium supplements (1.3 % p.a, 95%CI +0.49, +2.17) and persistent self-reported change in physical activity levels (0.7% p.a., 95%CI +0.22, +1.22).

**Conclusion:**

Individualized BMD feedback combined with a minimal educational intervention is effective at increasing hip but not spine bone density in premenopausal women. The changes in behavior through which this was mediated are potentially important in the prevention of other diseases, thus measuring BMD at a young age may have substantial public health benefits, particularly if these changes are sustained.

## Background

Osteoporosis is a major and growing public health problem, particularly in women[1], with an estimated 10 million people already having osteoporosis in the US and 18 million more having low bone mass[2]. In the United States, the direct financial costs alone of osteoporosis are estimated at $10 to $15 billion annually. Low bone mineral density (BMD) is a major risk factor for osteoporotic fracture[3]. BMD in later life is a function of peak bone mass and the rate of subsequent bone loss[4]. Although bone mineral density is lost most rapidly post-menopausally, it has also been shown that premenopausal women have significant age-related BMD loss [5-7] and that premenopausal bone mass contributes to fracture risk in later life[8].

Modifiable risk factors for low BMD include low calcium intake, smoking and low levels of physical activity[2]. Limited information is available currently on how to influence these risk factors in premenopausal women. Three uncontrolled studies have suggested that BMD screening with feedback of results combined with an information leaflet increases self-reported osteoporosis preventive behavior change at twelve months, in women aged 30 to 80 years[9] and in exclusively premenopausal women[10,11]. In particular, greater changes were reported in women with low BMD. However, there have been no longer-term follow-up studies or studies of the effect, if any, of these behavior changes on BMD. Similarly, studies of educational interventions in pre-menopausal women that have not included a bone density feedback component [12-15] have been short-term and have not studied effects of behavior change on BMD.

The aim of this study was to determine the effects of individualized bone mineral density (BMD) feedback and two different educational interventions on osteoporosis preventive behavior and 2-year change in BMD in pre-menopausal women. Specifically, we aimed to test the following hypotheses:

1. Women are more likely to change calcium intake and physical activity if their BMD is low.

2. Group education (in the form of the Osteoporosis Prevention and Self Management course) will be more efficacious at changing these lifestyle behaviours than an information leaflet alone.

3. Bone density feedback and educational intervention have independent effects on behavior and BMD change.

4. Women who improve their physical activity or dietary calcium intake will have a change in bone mass over 2 years that is 0.34–0.54% per annum better (depending on site and lifestyle factor) than those who do not alter their behaviour.

## Methods

**Figure 1:**
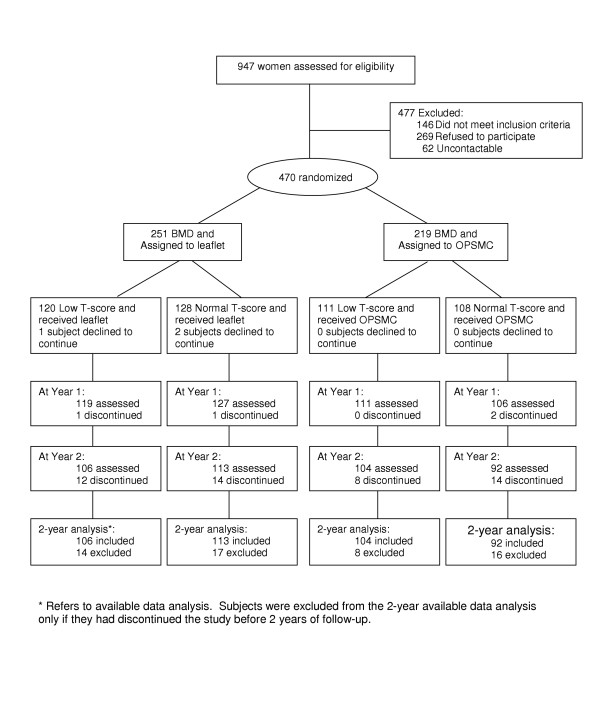
Flow of subjects through the trial.

The study was performed in Southern Tasmania in 2000–2003. The population of the region is predominantly Caucasian and as at June 1999 numbered 194 389 with 28 839 women aged between 25 and 44 years of age[16]. Subjects were selected at random with equal probabilities of selection using the Tasmanian Electoral Roll as the sampling frame. Voting is compulsory in Australia and this register of electors provides a comprehensive population listing that is estimated to be 95% complete for persons of these ages [17]. Subjects were excluded if they had previously had measurement of bone densitometry, had thyroid disease, renal failure, malignancy or rheumatoid arthritis, had a history of hysterectomy, were on hormone replacement therapy or were pregnant or planning pregnancy within 2 years of study entry, or were lactating. A total of 146 subjects were excluded on these grounds (Figure 1). Ethics approval was obtained from the Royal Hobart Hospital Ethics Committee and all subjects gave written informed consent.

### Interventions

Details of the interventions used have previously been published[18], but a brief description is given below.

### Bone density feedback

Subjects had their bone mineral density at the spine and hip, together with body composition, measured at baseline and at 2 years (Hologic QDR2000, Waltham, MA). At baseline, those with a mean T-score at spine and hip of greater than or equal to 0 received a letter informing them that they were not at a higher risk of fracture in later life, whereas those who had a mean T-score of less than 0 were informed that they were at higher risk. This was based on the observation that those in the lower half of the BMD distribution have threefold higher fracture risk both in later life and in the early postmenopausal period [19] suggesting that bone density tracks throughout life as has been recorded in children[20]. Though this cut off is based on women older than in the current study, there is evidence that has led to the proposition that bone mass tracks throughout life [21] so that women who are in the lower BMD range premenopausally may go on to have lower BMD postmenopausally. T-scores were used rather than Z-score as the emphasis of the individual feedback was on fracture risk in later life, as predicted by T-scores, rather than current fracture risk. However, given the young age of our sample, the distribution of Z-scores and T-scores was very similar.

### Education

Subjects were randomized, before their BMD result was known, to receive one of two interventions: an information leaflet produced by Osteoporosis Australia "Understanding Osteoporosis"; or the Osteoporosis Prevention and Self-management Course (OPSMC). Prior to recruitment, a random number drawn from a (0,1) uniform distribution (using the random number generator in SAS) was allocated to a participant number (which were consecutive integer values starting at 1). The random numbers were rounded to 0 if less than 0.5, or 1 if greater than or equal to 0.5. The 0 or 1 produced the assignment to one of the two intervention groups. Participants were allocated a participant number on recruitment. While there was no allocation concealment, allocations were implemented sequentially for each participant number with no variations to the order in which the numbers were assigned.

The OPSMC is a chronic disease self-management course based on the work of Lorig[22,23] developed by the Arthritis Foundation of Victoria and utilized by Osteoporosis Australia. This small group patient education program aims to increase knowledge, improve confidence and awareness and self-management of osteoporosis prevention with an emphasis on promoting appropriate lifestyle change. There were a maximum of 16 subjects per group and sessions were held at regular intervals for 2 hours per week for four weeks. Participants randomized to the leaflet intervention received the same osteoporosis information leaflet as in our previous study [11]. The leaflet provides a description of osteoporosis, an overview of the role of lifestyle factors such as diet, exercise and smoking and outlines ideal levels of calcium intake and exercise. It was considered unethical to provide no educational information to subjects so the leaflet was given as minimal information. Participants randomized to the leaflet intervention received their BMD feedback with the leaflet and participants randomized to the OPSMC received their BMD feedback at the first session of the course.

Primary outcome measures were bone mineral density at the femoral neck and lumbar spine, and the physical activity and calcium intake measures described below.

Usual calcium intake was assessed at yearly intervals by a short food frequency questionnaire (FFQ) designed specifically to measure calcium intake, with a reference period of the last 12 months. The FFQ has been validated in Caucasian Australian women against 4 day weighed dietary records. The correlation between methods for estimated calcium intake was high (r = 0.79, p = 0.001)[24]. Calcium content of food categories was assigned using Australian food composition tables[25], and usual calcium intake estimated in mg/day. Information on whether respondents were taking calcium supplements was also obtained by questionnaire. Respondents were classified as taking calcium supplements if they reported taking a supplement containing calcium alone or as a main ingredient, and at a frequency of not less than 4 times per week.

Physical activity was assessed in three ways. Energy expenditure and sports participation was assessed annually by a questionnaire validated in US adolescents[26] which has been modified for Tasmanian conditions and used previously in women in this age group where it was associated with bone mass at the femoral neck[27]. In this questionnaire, strenuous physical activity levels were assessed by how many days in the last 14 the subjects reported performing at least 20 minutes of strenuous exercise and light exercise, measured in five categories (1 = 0 days, 2 = 1–2 days, 3 = 3–5 days, 4 = 6–8 days, 5 = 9 or more days). Muscle strength and endurance fitness was assessed at baseline and two years. Muscle strength was assessed by dynamometry in the lower limb. The intraclass correlation coefficient for lower limb strength was 0.88 (95% CI 0.84, 0.92) at baseline and 0.82 (95% CI 0.73, 0.92) at 2 years. Endurance fitness was assessed by bicycle ergometer where physical work capacity at 170 beats per minute was estimated by progressively increasing sub maximal workloads[28]. This measure correlates well with treadmill assessment of VO_2 _max[29].

Other factors measured at baseline and 2 years were: height by stadiometer (The Leicester height measure, Invicta Plastics Ltd, Oadby, England) and weight by a single set of calibrated scales (Heine, Dover NH USA). Body mass index was calculated (kg/m^2^). Questionnaire assessment was made of smoking history (current/former/never, cigarettes per day, age at uptake, age at ceasing), breastfeeding history (ever breastfed, time since last breastfeeding), number of children, family history of osteoporosis and/or fracture, as well as fracture history in the subject, education level (4 point scale: less than grade 10, up to grade 10, completed grade12, tertiary), employment status of main financial provider in the household (employed or unemployed), hours of employment of the respondent (0, less than or equal to 20 or >20 hours per week) and marital status.

At one and two years, subjects were followed up by mail, using the questionnaires described above, and by asking a series of yes/no questions to assess self-reported change in smoking, dietary calcium intake, calcium supplement use and physical activity.

As the subjects were aware of both their BMD status and of the intervention they received, blinding of assessors was not attempted.

### Statistical analysis

All statistical analyses were based on the a priori hypotheses above. We performed statistical power calculations which indicated that we had power of 0.8 (α = 0.05 (two-tailed)) to detect clinically meaningful changes in calcium intake and physical activity, and differences in BMD of 1% or better.

Paired t-tests were performed to compare baseline and 2 year femoral neck and lumbar spine BMD for the whole study sample, and one-way ANOVA to compare BMD within the four intervention groups. Simple linear regression and one-way ANOVA were used for continuous and categorical measures respectively to examine the relationships between BMD change and intervention groupings, and changes in osteoporosis preventive behaviors. Multiple regression modeling, including potential confounders, was then used to examine the relationships between T-score group and educational intervention and BMD change as well as changes in behavior and BMD change, at both the femoral neck and lumbar spine. A number of behaviors were measured in different ways, for example changes in physical activity, smoking, calcium intake and calcium supplement use were measured by detailed questionnaires, as well as by simple self-report (change in behavior – yes/no). In our modeling, we assessed associations between changes in BMD and each method of measuring a behavior separately. In multivariate analysis, for behaviors measured in more than one way, we reported the effects using the using most objective measure e.g. FFQ assessment of calcium supplement use and dietary calcium intake rather than simple self-report of changes in calcium supplement use and dietary calcium. For simple self-reported physical activity change and smoking change, which were measured at both 1 and 2 years, we reported on effects of persistent improvement i.e. improved behavior reported at both 1 and 2 years.

The analysis was performed in 3 ways

(1) by available data analysis, which included all subjects who reached 2 years of follow-up;

(2) by intention to treat, in which all randomised individuals were included in the analysis. We imputed missing data at 2 years using the method of last observation carried forward [30] for measured variables, and imputed no change for self-reported behavioral change variables; and

(3) by per protocol analysis defined in two ways: firstly, by whether subjects attended at least one of four educational session of the OPSMC, and secondly, by whether they attended all four OPSMC sessions.

A sensitivity analysis was also performed omitting subjects with a baseline T-score < -2.5, which may lead to pharmacological treatment for osteoporosis in our location. No adjustment was performed for multiple comparisons.

All analyses were performed in Stata version 7 (Stata Corporation, Texas, USA). Statistical significance was set as p < 0.05 (two-tailed).

## Results

A total of 470 women (response rate of 64%) were recruited. Of these, 415 (88%) reached final follow-up. There were no statistically significant differences in baseline demographics and proportions of participants receiving the OPSMC and low T-score feedback between those completing the study and those withdrawing (data not shown).

Table 1 shows the baseline characteristics of women in each intervention group. As expected, women in the low T-score categories, were shorter and lighter than those in the high T-score category. There was a trend (p = 0.05) for a greater proportion of women in the low T-score groups to be taking calcium supplements, but the proportion in both groups was small. The groups were otherwise similar. Only three subjects had a femoral neck or lumbar spine T-score of less than -2.5.

Across the whole study sample, from baseline to 2 years there was a 1.1% p.a. (95%CI +0.9, +1.4) increase in femoral neck BMD from baseline to 2 years and no change in lumbar spine BMD (+0.09 %p.a., 95%CI -0.06, +0.20).

**Figure 2:**
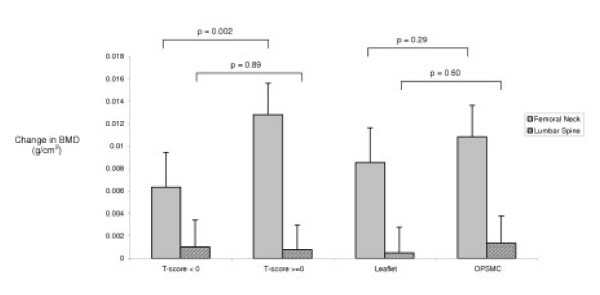
Change in BMD by T-score group and educational intervention over 2 years. P-values are comparisons between T-score and between educational groups. Data is presented as mean and upper 95^th ^CI.

Table Two compares BMD changes between low and high T-score groups and between the leaflet and OPSMC educational intervention groups. Subjects in the low T-score group had a greater percentage rate of change in femoral neck BMD as well as higher absolute change (Figure 2). By comparison, there was no difference in the rate of change in femoral neck BMD between the leaflet and OPSMC groups. There were no differences in rates of lumbar spine BMD change between either T-score or education groups.

**Figure 3:**
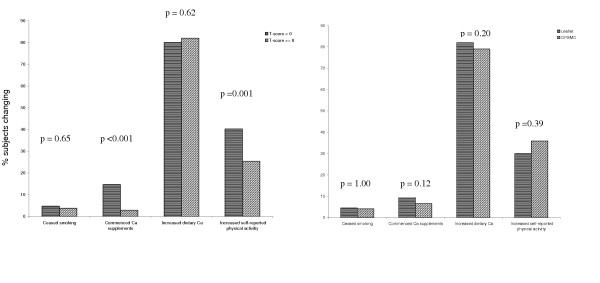
Effect of intervention on change in osteoporosis preventive behaviors (a) by T-score(b) by educational intervention. P-values are for comparison of proportion of subjects changing each behavior between T-score groups and between educational intervention groups at two years. Significant differences in behavior change between groups were only observed for calcium supplement use and self-reported physical activity between T-score groups. The type of educational intervention did not affect the proportion of subjects changing osteoporosis preventive behaviors.

**Figure 4:**
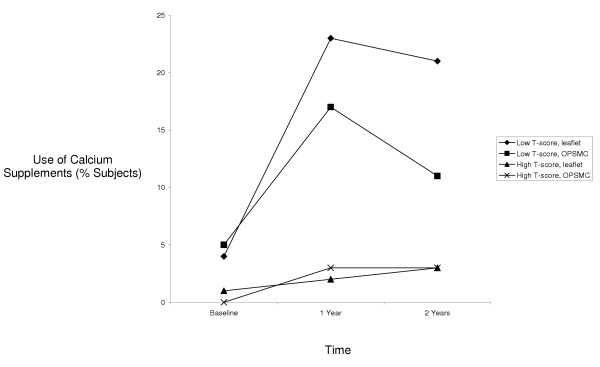
Calcium supplement use by intervention group. This shows changes in calcium supplement use by subjects in each of the four intervention groups over the study period. Only those intervention groups that included feedback of low T-score had significant increases in calcium supplement use.

Figure 3 shows differences in osteoporosis preventive behaviors at two years by T-score group and by educational intervention. A greater proportion of subjects in the low T-score group commenced taking calcium supplements (as measured by FFQ) and reported changes in physical activity than in the high T-score group, but there were no differences between education groups. Levels of dietary calcium intake and smoking cessation were similar across both T-score and educational groups. Figure 4 demonstrates that T-score group, not educational intervention is the main determinant of commencing use of calcium supplements. There were no significant differences in changes in strenuous activity levels, average leg strength change, and change in work capacity between T-score groups or between educational groups (data not shown).

**Figure 5:**
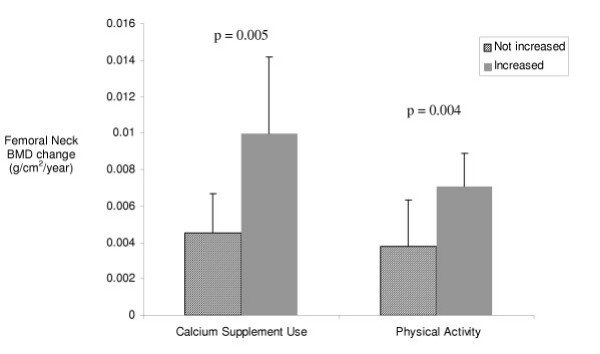
Mean absolute change in femoral neck BMD per year for women who did or did not increase calcium supplement use or physical activity. Data is presented as mean and upper 95^th ^CI. P-values are for differences between women who changed and who did not change each behavior.

Table 3 documents the associations between changes in femoral neck and lumbar spine BMD and changes in osteoporosis preventive behaviors. In univariate analysis there were positive associations between change in femoral neck BMD and calcium supplement use (whether measured by FFQ or self-reported behavior change) and physical activity (by self-report but not questionnaire assessment). Figure 5 shows the absolute differences in femoral neck BMD in those who did and did not change calcium supplement use and self-reported physical activity. In multivariate analysis these associations persisted. At the lumbar spine, there were no significant associations in univariate analysis. However, a positive association between change in work capacity and lumbar spine BMD change was significant in multivariate analysis.

All analyses were repeated after imputation of data for participants lost to follow-up (i.e. an intention to treat analysis) and the associations reported above were not altered (data not shown). The associations were unchanged also when analysis was repeated for actual OPSMC participation rather than available data analysis (Table 2) or if subjects with a baseline T-score of less than or equal to -2.5 were omitted (data not shown).

## Discussion

This study demonstrates that bone density feedback with minimal patient education in premenopausal women is effective at increasing hip but not lumbar spine bone density and that this effect appears to be mediated by changes in physical activity and calcium supplement usage. However, group education had no additional effect over a simple information leaflet.

Women receiving feedback of a low T-score result were more likely to commence calcium supplement use and to report changes in physical activity, as well as to have greater increases in femoral neck BMD, compared to women with a high T-score.

Notably, the magnitude of these BMD changes, which occurred outside the setting of randomized controlled trials of interventions of calcium supplements and exercise, is similar to that obtained within such trials. Specifically, the magnitude of the effect on femoral neck BMD of self-reported physical activity change in our study was +0.7% p.a. and for calcium supplement use was +1.3 % p.a., which compared to treatment effects in premenopausal women reported for randomized controlled trials of exercise interventions of 0.9 % p.a[31] and for calcium supplement use of 1% p.a. respectively[32]. The ability to achieve these changes with relatively simple interventions has major potential public health benefits for osteoporosis prevention and fracture reduction in later life. For example, in drug trials a change in BMD of 5% with bisphosphonates leads to a 50% decrease in fracture risk[33] while regular walking is associated with a 50% decrease in hip fracture risk[34]. The resulting behavior changes could also be potentially beneficial for prevention of other chronic diseases whose incidence could be reduced by increased calcium intake and/or increased physical activity, such as cardiovascular disease, obesity and diabetes mellitus.

Interestingly, the results suggest that an intervention as intensive as the OPSMC is no more effective at increasing BMD than a relatively simple and inexpensive educational intervention such as an information leaflet in this age group. The OPSMC was modeled upon a chronic disease self-management course for arthritis, which has been shown to be modestly effective in symptomatic populations at reducing health care utilization and improving health status [22,23]. The lack of increased effect by the addition of the OPSMC in this group of healthy, asymptomatic women is possibly due to differences in motivation between women undertaking a chronic disease self-management course who actually have a symptomatic condition, compared with a course aimed at preventing a chronic disease in healthy women. Further research is required to address the OPSMC's role in the management of other populations of women such as women with existing osteoporosis and/or fracture.

The findings that all intervention groups showed increased femoral neck BMD and that there was essentially no change in lumbar spine BMD are noteworthy given observational data for this age group. While there is no natural history data available in our geographic location, longitudinal studies examining the natural history of BMD loss in other Caucasian populations have been consistent with there being onset of bone loss in the pre-menopausal period[5,6] with one study reporting annual loss of BMD at the femoral neck of about 0.3 % of baseline BMD p.a., and bone loss potentially beginning as early as age 24[7]. The onset of lumbar spine bone loss was estimated at 38–39 years, with the maximum annual loss being 0.5% p.a. If premenopausal bone loss can be reduced, or potentially reversed, then this has important implications for the long-term prevention of osteoporosis and fracture. The ability to achieve this with a relatively simple intervention i.e. bone mineral density feedback with an information leaflet, has major potential public health benefits for osteoporosis prevention. However, confirmation of long-term benefits and assessment of the cost effectiveness of the intervention needs to occur before any recommendation for implementation at a population level is made.

The associations between changes in BMD and physical activity varied by site of BMD measurement, and by the physical activity measure used. Simple self-report of physical activity change was positively associated with change in femoral neck BMD, but not lumbar spine BMD. However, there was no association between changes in other physical activity measures and femoral neck BMD. Change in endurance fitness was associated with rate of change of lumbar spine but not femoral neck BMD. Changes in femoral neck BMD in exercise interventions without accompanying increases in muscle strength have been observed previously in premenopausal women[35]. Different types of exercise can also cause different BMD responses in the lumbar spine and femoral neck BMD[36,37]. Variation in the types of activity captured by simple self-report and the site dependency of the BMD effects of different activities may account for the differences in associations seen at the lumbar spine compared to femoral neck. Alternatively, the femoral neck may be more susceptible to lifestyle interventions in premenopausal women than the lumbar spine. The difficulty of capturing all dimensions of physical activity by questionnaire is well-known[38,39], particularly non-leisure activities in women[40]. Simple self-report may have captured changes in incidental activity, not detected by other measures, which may have caused increases in femoral neck BMD. The use of pedometry or accelerometry to assess physical activity changes in future research may assist with elucidating the reasons behind the discrepant results.

There were no significant associations between femoral neck or lumbar spine BMD and changes in smoking habits or dietary calcium intake. The number of women who persistently ceased smoking over the study period was small (n = 20), so the study had insufficient power to detect a meaningful difference in BMD. The difficulties of finding associations between calcium intake and BMD are well known[41]. The measurement error inherent in FFQ assessment of dietary intake, combined with variations between database derived nutrient contents food and actual content, and variations in the bioavailability of calcium in individuals, all make the identification of associations outside of randomized controlled trials of calcium intake problematic. In contrast, ascertainment of calcium supplement usage may have less error, thus making it easier to detect an association with calcium supplement use, as we found in this study.

This study has a number of potential limitations. While the sample was randomly selected, selection bias is possible due to the 64% response rate. The proportion of current smokers in the sample is lower than the Tasmanian prevalence of daily smoking in females aged 25–44 years in 1998 of 29% [16]. However, the wide spread of education levels and the unemployment rate approximates the overall population figures for these socioeconomic factors. We have previously reported that calcium intake in this cohort is only slightly higher than that reported in a Southern Tasmanian sample of women who were selected using criteria that resulted in a cohort with high levels of smoking[27] suggesting that while there is a potential for selection bias towards a healthy cohort, the effects of this are minor and that the results of this study are likely to be generalisable to healthy Caucasian women in the 25 to 44 year age range. Regression to the mean may account for some of the observed change in femoral neck BMD. It would not affect the associations between BMD feedback and behavior change, nor the associations between behavior change and BMD change. The extent of regression to the mean decreases as the correlation between pre and post intervention values increases. In this case, the correlation between baseline and follow-up femoral neck BMD was high (r = 0.87) making the likelihood of regression to the mean low[42,43]. Furthermore, if there were significant regression to the mean, we would expect to see a decrease in the high T-score group and a similar effect at the lumbar spine, which was not the case. The period of follow up for this study question is greater than that previously reported at 2 years. However, the effect of the remodeling transient which results from increased calcium intake is greatest in the 6 to 18 months after increasing calcium intake[44] so it is not possible to determine whether the effect of calcium on BMD over the two years of the study is indicative of long-term gain in steady-state bone mass or is due to the remodeling transient. Longer-term follow-up is needed to confirm any lasting positive effects on BMD from ongoing calcium supplement use. Even longer follow-up, in a much larger sample, would be needed to detect any positive effects on fracture incidence. We chose not to have an intervention group receiving BMD feedback alone without any other educational intervention. This decision was made because previous studies involving BMD feedback affecting behaviour had included leaflet information [9-11] and there was therefore an argument that supplying a minimal amount of information would be the minimum intervention that would be acceptable both practically and ethically. While this limits the ability to state categorically which effects were due to the BMD feedback and which the leaflet, the variability between T-score subgroups suggests that BMD feedback was the more important component of the intervention. This is consistent with the results of the only randomized controlled trial of written information alone, which found no changes in behaviour from the intervention [14]. We performed additional analysis by intention to treat to assess the likelihood of loss to follow-up influencing these results, and found that the observed associations remained and were not materially different in magnitude. Lastly, to ensure the effects seen were not confounded by treatment for osteoporosis, we performed the analysis omitting the those subjects known to have been treated as well as those subjects whose T-score would have qualified them for treatment, i.e. less than -2.5 at any site. This did not substantially alter the study findings.

## Conclusion

Individualized BMD feedback combined with a minimal educational intervention is effective at increasing hip but not spine bone density in premenopausal women. The changes in behavior through which this was mediated are potentially important in the prevention of other diseases, thus measuring bone density at a young age may have substantial public health benefits, particularly if these changes are sustained.

## Competing interests

The author(s) declare that they have no competing interests.

## Authors' contributions

GJ was involved in study design, supervised fieldwork and was involved in drafting the manuscript. BO was involved in study design and was involved in drafting the manuscript. SF was involved in study design and the adaptation and implementation of the OPSMC for the project and in revising the manuscript. LD performed data collection and provided input into the drafting of the manuscript. MR was involved in study design, particularly of the nutritional assessment component, performed the analysis of the food frequency questionnaires and provided input into the drafting of the manuscript. TW performed data cleaning and analysis and was primarily responsible for drafting the manuscript. All authors read and approved the final manuscript.

## Pre-publication history

The pre-publication history for this paper can be accessed here:



## Figures and Tables

**Table 1:** Comparison of baseline characteristics of each intervention group.

	T-score ≦ 0 and leaflet (n = 128)^a^	T-score ≦ 0 and OPSMC (n = 108)	T-score<0 and leaflet (n = 119)	T-score<0 and OPSMC (n = 112)
Age (years)	37.9 (5.3)	37.4 (5.8)	38.4 (5.0)	37.4 (5.3)
Height (cm)	164.2 (6.4)	164.2 (5.6)	162.7 (6.5)	161.5 (6.5)
Weight (kg)	73.9 (15.2)	75.3 (13.6)	65.4 (10.0)	63.5 (10.8)
BMI (kg/m^2^)	27.5 (5.7)	27.9 (4.4)	24.8 (4.1)	24.4 (4.0)
Femoral Neck BMD (g/cm^2^)	1.01 (0.09)	1.03 (0.11)	0.83 (0.08)	0.84 (0.12)
Lumbar Spine BMD (g/cm^2^)	1.16 (0.09)	1.18 (0.10)	0.99 (0.07)	0.99 (0.08)
Calcium Intake (mg/day)	805 (429)	795 (406)	753 (368)	803 (389)
Calcium Supplement Use, %	1	0	3	4
Work Capacity (W)^a^	2.3 (0.7)	2.3 (0.7)	2.3 (0.7)	2.3 (0.6)
Average Leg Strength (kg)^b^	1.3 (0.4)	1.3 (0.4)	1.4 (0.4)	1.4 (0.4)
Median Strenuous Activity Level	3	3	3	3
Currently smoking, %	20	16	18	13

^a ^mean (SD) unless otherwise stated

^b ^per kg body weight

**Table 2:** Effect of bone density feedback and group education on BMD change.

	Univariate β (95% CI)		Multivariate β^a,b ^(95% CI)		Multivariate β^a,c ^(95% CI)		Multivariate β^a,d ^(95% CI)	
*Femoral Neck BMD change (% p.a.)*								
T-score (<0 vs >0)	**+0.93**	**(+0.46,+1.4)**	**+0.86**	**(+0.39,+1.34)**	**+0.85**	**(+0.38,+1.30)**	**+0.86**	**(+0.39,+1.34)**
OPSMC v leaflet	+0.22	(-0.26,+0.70)	+0.14	(-0.32,+0.62)	+0.38	(-0.20,+0.96)	+0.19	(-0.29,+0.68)

*Lumbar Spine BMD change (% p.a.)*								
T-score (<0 vs >0)	+0.02	(-0.29,+0.32)	-0.01	(-0.32,+0.30)	-0.02	(-0.32,+0.30)	-0.01	(-0.32,+0.30)
OPSMC v leaflet	+0.10	(-0.21,+0.40)	+0.09	(-0.21,+0.40)	+0.21	(-0.16,+0.59)	+0.02	(-0.30,+0.34)

^a^adjusted for other items in column, age, and difference in weight and height between baseline and 2 years.

^b ^available data analysis

^c^per protocol analysis defined as subjects attending all OPSMC sessions

^d^per protocol analysis defined as subjects attending at least one OPSMC session

**Table 3:** Effect of behavior change on BMD change

*Femoral Neck BMD change (% p.a.)*	Univariate β (95% CI)		Multivariate β^a ^(95% CI)	
Commenced calcium supplements	**+1.37**	**(+0.54, +2.2)**	**+1.33**	**(+0.49, +2.17)**
Calcium intake change (per 100 mg)	-0.03	(-0.07, +0.02)	-0.03	(-0.08, +0.01)
Persistent smoking cessation	+0.21	(-0.9, +1.34)	-0.04	(-1.16, +1.08)
Persistent self-reported physical activity change	**+0.77**	**(+0.28, +1.3)**	**+0.72**	**(+0.22, +1.22)**
Persistent increase in strenuous activity	+0.21	(-0.35, +0.76)	+0.11	(-0.45, +0.67)
Change in work capacity (per W)	-0.10	(-0.52, +0.32)	-0.06	(-0.48, +0.36)
Change in leg strength (per SD)	+0.02	(-0.22, +0.26)	+0.02	(-0.22, +0.26)
*Lumbar Spine BMD change (% p.a.)*				
Commenced calcium supplements	+0.08	(-0.45, +0.62)	+0.18	(-0.37, +0.73)
Calcium intake change (per 100 mg)	0.00	(-0.03, +0.03)	-0.01	(-0.04, +0.02)
Persistent smoking cessation	+0.21	(-0.50, +0.93)	+0.11	(-0.62, +0.85)
Persistent self-reported physical activity change	-0.10	(-0.42, +0.21)	-0.05	(-0.38, +0.28)
Persistent increase in strenuous activity	-0.17	(-0.52, +0.18)	-0.16	(-0.53, +0.21)
Change in work capacity (per W)	+0.24^b^	(-0.02, +0.51)	**+0.31**	**(+0.03, +0.59)**
Change in leg strength (per SD)	+0.07	(-0.08, +0.23)	+0.05	(-0.10, +0.22)

^a^adjusted for other items in column, age, and difference in weight and height between baseline and 2 years. ^b^p = 0.07
